# Evaluating KRAS-Associated Responses to Sulfasalazine and 5-Fluorouracil in Colorectal Cancer Using Integrated 2D and PEGDA Microwell-Based 3D Tumor Models

**DOI:** 10.3390/ijms27146238

**Published:** 2026-07-13

**Authors:** Mehrdad Bandegi, Ezgi Biltekin, Yasemin M. Akay, Metin Akay

**Affiliations:** Department of Biomedical Engineering, Cullen College of Engineering, University of Houston, Houston, TX 77204, USA; mbandegi@cougarnet.uh.edu (M.B.); ymakay@uh.edu (Y.M.A.)

**Keywords:** colorectal cancer, KRAS/MAPK, sulfasalazine, 5-fluorouracil, PEGDA microwells, 3D spheroids, drug resistance, combination therapy

## Abstract

Colorectal cancer (CRC) is a major cause of cancer-related mortality among adults younger than 50 years of age, and many tumors show incomplete response or develop resistance to 5-fluorouracil (5-FU)-based chemotherapy. Therefore, new therapeutic approaches that improve CRC sensitivity to existing chemotherapeutic agents are needed. *KRAS*-associated signaling contributes to CRC growth, metabolic adaptation and treatment resistance. In this study, we investigated whether sulfasalazine (SSZ), a U.S. Food and Drug Administration (FDA)-approved anti-inflammatory drug, could enhance the response of CRC cells to 5-FU and modulate KRAS/mitogen-activated protein kinase (MAPK)-associated signaling. Public dataset analysis using cBioPortal, Kaplan–Meier Plotter and DepMap showed that *KRAS* is commonly altered in CRC. The analysis also showed that higher KRAS expression was associated with shorter overall survival in 1061 CRC patients, while CRC cell-line models demonstrated *KRAS* dependency. In 2D cultures, both *KRAS*-mutant HCT116 and *KRAS*-wild-type RKO cells showed lower cell viability, reduced colony formation and decreased KRAS expression after SSZ treatment. In 3D cultures, exposure to SSZ reduced early spheroid formation, both as a single treatment and when combined with 5-FU. In established spheroids, SSZ-containing treatments affected cell viability, spheroid growth and morphology, with the most noticeable suppressive effect observed in RKO aggregates. SynergyFinder+ dose-matrix analysis identified dose ranges where SSZ and 5-FU showed additive-to-synergistic effects, leading us to select 600 μM SSZ with 25 μM 5-FU for further validation. Western blot results from PEGDA microwell-derived 3D spheroids showed that SSZ + 5-FU treatment reduced KRAS expression and affected KRAS/MAPK-related signaling. This effect was more pronounced in RKO cells, where downstream pathway suppression was stronger. The combination treatment also increased apoptosis-associated PARP cleavage. At the same time, it reduced Cyclin D1 and GPX4 protein levels and changed the expression of stemness-related markers, including ALDH1A3, CD44, and CD133. Together, these results support SSZ as a candidate repurposed adjuvant that may improve the response to 5-FU in CRC spheroid models and support the use of PEGDA microwell-based 3D platforms for testing combination therapy approaches.

## 1. Introduction

Colorectal cancer (CRC) is a major public health burden, with an estimated 158,850 new cases and 55,230 deaths expected in the United States in 2026, which makes it the second leading cause of cancer-related death overall and the leading cause of cancer death among adults younger than 50 years [1,2,3]. Fluoropyrimidine-based chemotherapy has improved CRC management, and 5-fluorouracil (5-FU) alone or in combination remains a backbone of chemotherapeutic treatment regimens. However, incomplete response and acquired resistance to 5-FU still limit durable treatment benefit in CRC [4,5]. Therefore, combination strategies targeting tumor survival mechanisms and improving the response to chemotherapy still play an important role in CRC research.

Kirsten rat sarcoma viral oncogene homolog (*KRAS*)-associated signaling is closely related to CRC progression and therapeutic resistance. Activation of *KRAS* alterations supports tumor-cell proliferation, survival, migration/invasion, and metastatic progression by maintaining downstream RAF/MEK/ERK and PI3K/AKT signaling [6,7]. *KRAS*-mutant CRC cells may also experience metabolic adaptations, including glutamine-dependent metabolic rewiring and changes in redox balance, to maintain persistent *KRAS* activation and potentially influence treatment response [8]. In parallel, resistance to 5-FU-based therapy involves multiple mechanisms, including altered drug metabolism, impaired apoptosis, cancer stem-like phenotypes, and DNA damage-repair pathways [5,9,10]. Although mutation-selective KRAS inhibitors have expanded treatment options for selected patients, broad *KRAS* targeting in CRC remains limited [11,12]. For this reason, approaches that reduce KRAS-associated growth signaling or exploit KRAS-linked stress vulnerabilities may provide useful combination strategies. In this study, we used public genomic datasets, survival-analysis data, and DepMap CRISPR gene-effect/dependency data from CRC cell lines to examine the clinical and functional relevance of KRAS-associated signaling in CRC tumorigenesis and patient survival.

Sulfasalazine (SSZ) is an FDA-approved anti-inflammatory drug used to treat inflammatory bowel disease and rheumatoid arthritis [13,14]. In gastric and colorectal cancer studies, SSZ has been investigated as a repurposed agent that acts by inhibiting system x_c−, the cystine/glutamate antiporter containing the light-chain subunit xCT/SLC7A11 [15,16]. The x_c− system provides cystine for cysteine production and glutathione synthesis, which are essential for antioxidant defense. Inhibition of this transporter can lead to decreased glutathione-dependent protection, enhanced oxidative stress and sensitivity of tumor cells to treatment-induced damage. In CRC, SSZ has been reported to sensitize tumor cells to cisplatin through glutathione-dependent mechanisms and to affect pathways linked to tumor growth and stemness-associated behavior [16,17]. However, to the best of our knowledge, the effect of SSZ on 3D spheroids in combination with 5-FU has not been investigated. These properties make sulfasalazine a suitable candidate to test as an adjuvant to 5-FU in CRC 3D models.

Three-dimensional tumor models provide information that conventional monolayer culture cannot fully capture. 3D spheroids better represent cell–cell contact, compact tumor architecture, diffusion-limited drug exposure and treatment-associated changes in morphology [18,19,20,21,22]. We previously used our poly(ethylene glycol) diacrylate (PEGDA)-based microwell systems successfully for 3D CRC and GBM spheroid formation [23,24].

In this study, we investigated whether SSZ can enhance the response to 5-FU in CRC models with different *KRAS* backgrounds. In 2D CRC cell models, SSZ as a single agent caused a dose-dependent reduction in KRAS expression, cell viability, and colony formation. We then tested SSZ and 5-FU in 3D models by assessing their combined effect on early spheroid formation and established 3D spheroid cultures, followed by SynergyFinder+ analysis of drug-interaction patterns [25,26]. Selected treatment conditions were further examined in PEGDA microwell-derived spheroids by Western blot analysis to link drug response to molecular regulation via KRAS/mitogen-activated protein kinase (MAPK) signaling, apoptosis-associated cleaved PARP/PARP signaling, redox defense-associated markers, proliferation and stemness-associated markers. We found that combined SSZ + 5-FU treatment reduced KRAS expression, altered KRAS/MAPK-associated signaling, increased apoptosis-associated PARP cleavage, decreased glutathione peroxidase 4 (GPX4) protein levels, reduced Cyclin D1-associated proliferative signaling and modulated stemness-associated markers, including aldehyde dehydrogenase 1 family member A3 (ALDH1A3), CD44, and CD133.

## 2. Results

### 2.1. KRAS Is Frequently Altered in Colorectal Cancer and High KRAS Expression Is Associated with Shorter Patient Survival

**Figure 1:**
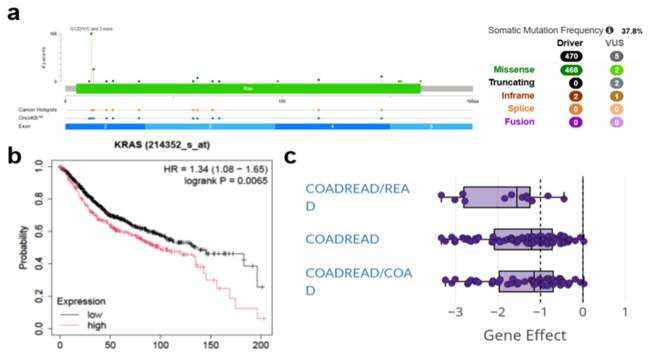
Integrated public dataset analyses support the clinical and functional relevance of *KRAS* in colorectal cancer. (**a**) cBioPortal analysis of a combined colorectal cancer cohort from three studies, including 1228 samples from 1225 patients, showed *KRAS* alterations in 473 patients, corresponding to 39% of queried patients. Mutation analysis showed a somatic *KRAS* mutation frequency of 37.8%, with most alterations classified as missense mutations and concentrated in canonical *KRAS* hotspot regions. (**b**) Kaplan–Meier Plotter overall survival analysis using the KRAS Affymetrix probe 214352_s_at in 1061 CRC patients showed that high KRAS expression was associated with shorter overall survival compared with low KRAS expression. The high-expression cohort had a median overall survival of 98 months, whereas the low-expression cohort had a median overall survival of 137 months, with hazard ratio (HR) = 1.34 and log-rank *p* = 0.0065. (**c**) DepMap CRISPR Gene Dependency analysis showed enriched *KRAS* dependency patterns in colorectal adenocarcinoma models. The COADREAD colorectal adenocarcinoma group included 96 models, with related subgroups including COAD colon adenocarcinoma models and READ rectal adenocarcinoma models. These data support the functional relevance of *KRAS* in CRC cell-line systems.

Public dataset analysis was performed to understand the clinical and functional importance of KRAS-associated signaling in CRC. cBioPortal analysis of a combined colorectal cancer cohort from three studies, including 1228 samples from 1225 patients, showed *KRAS* alterations in 473 patients, corresponding to 39% of queried patients [27,28]. Mutation analysis showed a somatic *KRAS* mutation frequency of 37.8%, with most alterations classified as missense mutations and concentrated in canonical hotspot regions (Figure 1a). Kaplan–Meier Plotter analysis using the Affymetrix probe (214352_s_at) showed that high KRAS expression was associated with shorter overall survival in 1061 CRC patients [29]. The high-expression cohort had a median overall survival of 98 months, compared with 137 months in the low-expression cohort, with an HR of 1.34 and a log-rank *p*-value of 0.0065 (Figure 1b). In addition, DepMap CRISPR Gene Dependency analysis showed enriched *KRAS* dependency patterns in 96 colorectal adenocarcinoma models, including COAD colon adenocarcinoma and READ rectal adenocarcinoma models (Figure 1c) [30]. Together, these public datasets highlight the importance of KRAS-associated signaling in CRC.

### 2.2. Sulfasalazine Suppresses CRC Cell Viability and Clonogenic Growth and Reduces KRAS Expression

**Figure 2:**
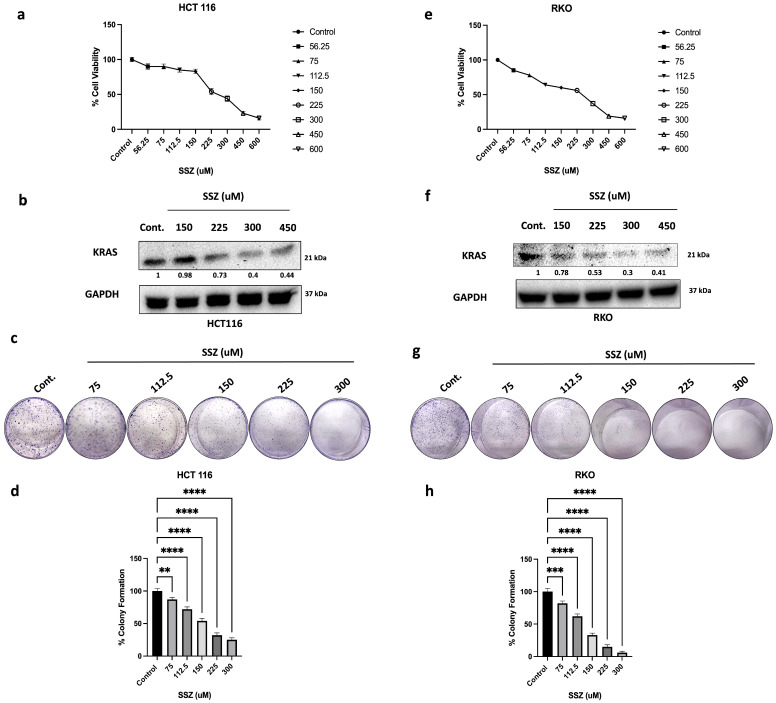
Sulfasalazine suppresses CRC cell viability, KRAS expression, and clonogenic growth in 2D HCT116 and RKO cultures. (**a**,**e**) Dose–response analysis after 72 h of sulfasalazine treatment showed concentration-dependent reduction in cell viability in HCT116 and RKO cells. Viability values were normalized to the DMSO control group. (**b**,**f**) Representative Western blot analysis showed reduced KRAS protein expression after treatment with selected sulfasalazine concentrations in both cell lines. GAPDH was used as the loading control. Densitometric values below the KRAS bands were calculated by normalizing KRAS band intensity to GAPDH and scaling each value to the untreated control group, which was set to 1.00. (**c**,**g**) Colony formation assays showed concentration-dependent suppression of clonogenic growth after sulfasalazine exposure in HCT116 and RKO cells. (**d**,**h**) Quantification of colony formation normalized to the control group. Data are presented as mean ± standard deviation (SD). Statistical significance was determined by one-way ANOVA. ** *p* < 0.01; *** *p* < 0.001; **** *p* < 0.0001.

To understand the effect of SSZ on short-term cell viability and long-term clonogenic growth, we performed 3-(4,5-dimethylthiazol-2-yl)-5-(3-carboxymethoxyphenyl)-2-(4-sulfophenyl)-2H-tetrazolium (MTS) and colony formation assays. HCT116 cells were used as a *KRAS*-mutant model, while RKO cells were used as a *KRAS*-wild-type model [31]. Three-day SSZ treatment resulted in dose-dependent inhibition of HCT116 and RKO cell viability. In HCT116 cells, viability remained relatively high at lower concentrations but decreased markedly at higher concentrations, with the strongest reduction observed at 450 and 600 µM (Figure 2a). The dose–response profile indicated an approximate IC_50_ range of 225–300 µM for HCT116 cells. RKO cells also showed dose-dependent inhibition, with a comparable approximate IC_50_ range of 225–300 µM; however, RKO cells showed a stronger decrease in viability at intermediate-to-high SSZ concentrations, particularly at 225–450 µM (Figure 2e). Western blot analysis showed that SSZ reduced KRAS protein expression in both HCT116 and RKO cells (Figure 2b,f). Colony formation assays confirmed that SSZ reduced clonogenic growth. In HCT116 cells, increasing SSZ concentrations progressively reduced colony formation, with strong suppression at 225 and 300 µM (Figure 2c,d). In RKO cells, colony formation decreased beginning at 150 µM and was nearly abolished at 225–300 µM (Figure 2g,h). These results show that SSZ had measurable single-agent activity in both CRC cell lines and that growth inhibition was accompanied by reduced KRAS expression under 2D conditions. The 2D dose–response data were used to guide the SSZ concentrations in the spheroid-formation and combination experiments.

### 2.3. Sulfasalazine and 5-Fluorouracil Suppress Early Spheroid Formation

**Figure 3:**
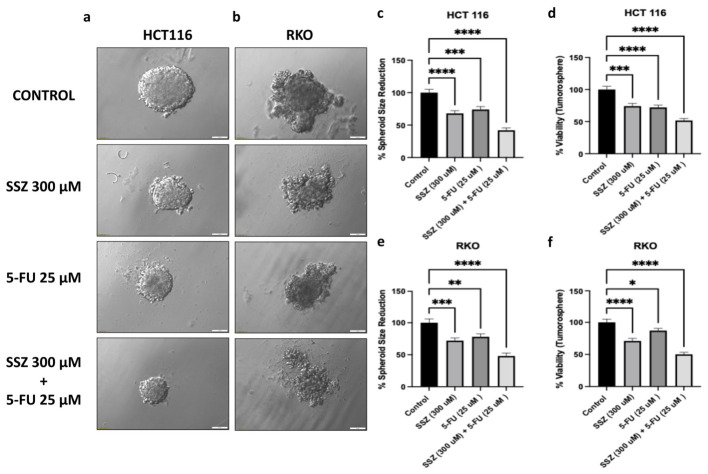
Combined sulfasalazine and 5-FU treatment suppresses spheroid formation and viability in CRC cells. (**a**,**b**) Representative bright-field images of HCT116 spheroids and RKO cell aggregates cultured under control, sulfasalazine (SSZ, 300 µM), 5-fluorouracil (5-FU, 25 µM), or combined SSZ + 5-FU treatment. (**c**,**e**) Quantification of spheroid/aggregate area showed treatment-dependent reduction in both HCT116 and RKO models, with the greatest decrease observed in the combination group. (**d**,**f**) Metabolic viability was measured using PrestoBlue HS and normalized to the control group. Data are presented as mean ± SD. Statistical significance was determined by one-way ANOVA followed by post hoc comparison. Scale bars = 100 µm. * *p* < 0.05; ** *p* < 0.01; *** *p* < 0.001; **** *p* < 0.0001.

The early-spheroid-formation assay was used to evaluate the effects of SSZ and 5-FU on the establishment of 3D CRC spheroid structures. Bright-field images were taken from three separate wells on Day 3 and assessed for spheroid/aggregate morphology and organization. The control group in the HCT116 model formed compact, rounded spheroids, whereas RKO control cells formed less uniform and more irregular aggregates on Day 3. SSZ treatment reduced HCT116 spheroid area and altered RKO aggregate organization. 5-FU also reduced HCT116 spheroid growth and changed the morphology of RKO aggregates. The combined SSZ + 5-FU treatment produced the most pronounced reduction in HCT116 spheroid area and the clearest reduction in RKO aggregate formation (Figure 3a,b).

Quantitative image analysis confirmed these imaging patterns. In HCT116 cells, SSZ 300 µM and 5-FU 25 µM alone reduced the relative spheroid area compared to the control, and the combination caused the greatest reduction (Figure 3c). In RKO cells, treatment reduced the relative area of cell aggregates, with the greatest reduction observed in the combination group (Figure 3e). Metabolic viability showed a similar pattern in both cell lines, with the lowest live-cell percentage observed in the combined SSZ + 5-FU group (Figure 3d,f). These results indicate that SSZ affected early 3D spheroid/aggregate formation and that the strongest reduction in spheroid/aggregate area and viability occurred when SSZ was combined with 5-FU.

### 2.4. Dose-Matrix Analysis Identified Additive-to-Synergistic Dose Combinations of Sulfasalazine and 5-Fluorouracil Treatments in Established Spheroids

**Figure 4:**
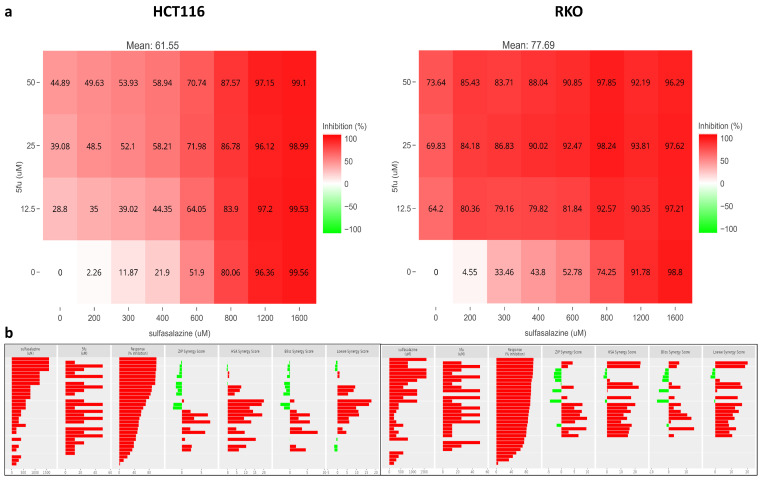
Matrix screens identify effective sulfasalazine + 5-FU dose regions in established CRC spheroids. (**a**) SynergyFinder+ inhibition heatmaps for sulfasalazine (SSZ; 0–1600 µM) × 5-fluorouracil (5-FU; 0–50 µM) in HCT116 and RKO spheroids. Higher inhibition is shown by deeper red color intensity. Mean inhibition across all combinations was 61.55% for HCT116 and 77.69% for RKO. (**b**) Waterfall plots display model-specific synergy scores for each combination using ZIP, HSA, Bliss, and Loewe models, focusing on additive-to-synergistic dose windows across the matrices.

To test the response of already-formed 3D CRC cultures under SSZ and 5-FU treatment, we treated established spheroids with a two-drug dose matrix of SSZ and 5-FU. Drug-interaction patterns were analyzed using SynergyFinder+. This experiment was conducted separately from the early-spheroid-formation assay because treatment began only after 3D structures had already formed on Day 3 of initial cell seeding. This design allowed us to evaluate whether SSZ could affect established spheroids rather than only interfere with their initial formation. The inhibition heatmaps showed dose-dependent responses in both HCT116 and RKO models (Figure 4a). Lower concentrations showed moderate inhibition in HCT116 spheroids, and the response increased with increasing SSZ concentrations, especially at 600–1600 µM. The mean inhibition across the HCT116 matrix was 61.55%. RKO cultures showed a stronger overall response, with a mean inhibition of 77.69%. In this model, several SSZ-containing dose ranges showed strong inhibition, particularly when higher SSZ concentrations were combined with 5-FU. We then evaluated drug-interaction scores using the ZIP, HSA, Bliss, and Loewe models (Figure 4b). ZIP estimates the response relative to an expected non-interaction effect, HSA compares the combination with the strongest single agent, Bliss evaluates the response based on independent drug effects, and Loewe evaluates the response based on dose additivity. The waterfall plots showed additive-to-synergistic dose windows in both HCT116 and RKO models. The response strength and distribution were different between the two cell lines, with RKO showing higher overall inhibition across the matrix. However, both models showed active combination areas on several synergy models. The results supported the selection of the SSZ and 5-FU dose pairs for imaging validation and molecular analysis.

### 2.5. Selected Doses of Sulfasalazine + 5-FU Show Additive to Synergistic Drug Interaction Effects in Established Spheroids

**Figure 5:**
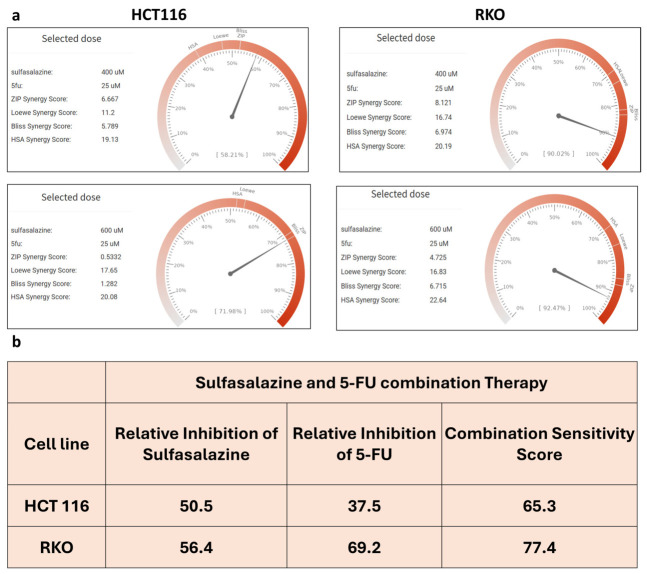
Dose selection and global sensitivity metrics for the sulfasalazine/5-FU combination in established CRC spheroids. (**a**) Selected-dose gauges from SynergyFinder+ showing active sulfasalazine/5-FU dose regions in HCT116 and RKO 3D cultures. ZIP, Loewe, Bliss, and HSA models were used to evaluate drug-interaction patterns. Based on these results, SSZ 600 µM + 5-FU 25 µM was selected for downstream validation and molecular analysis. (**b**) Relative inhibition of each single agent and the combination sensitivity score for each cell line. In HCT116, SSZ alone showed 50.5 relative inhibition, 5-FU alone showed 37.5 relative inhibition, and the combination sensitivity score was 65.3. In RKO, SSZ alone showed 56.4 relative inhibition, 5-FU alone showed 69.2 relative inhibition, and the combination sensitivity score was 77.4. In both models, the combination showed stronger activity than SSZ alone.

Selected-dose analysis was used to identify SSZ and 5-FU combinations with strong activity in established CRC spheroids. SynergyFinder+ selected-dose gauges showed additive-to-synergistic dose regions in both cell lines, although the response patterns differed between HCT116 and RKO. In HCT116, selected combinations produced ZIP synergy scores from 0.53 to 6.67, Loewe scores from 11.2 to 17.65, Bliss scores from 1.28 to 5.79, and HSA scores from 19.13 to 20.08. In RKO, selected combinations produced ZIP scores from 4.73 to 8.12, Loewe scores from 16.74 to 16.83, Bliss scores from 6.72 to 6.97, and HSA scores from 20.19 to 22.64. These values indicate additive-to-synergistic interaction patterns across the selected dose regions, with the strongest positive scores mainly observed in the HSA and Loewe models. Global sensitivity metrics also supported the activity of the combination (Figure 5b). In HCT116, SSZ alone showed a relative inhibition of 50.5, 5-FU alone showed a relative inhibition of 37.5, and the combination sensitivity score was 65.3. In RKO, SSZ alone showed a relative inhibition of 56.4, 5-FU alone showed a relative inhibition of 69.2, and the combination sensitivity score was 77.4. In both cell lines, the combination sensitivity score was higher than that of SSZ alone. Based on these results, SSZ 600 µM with 5-FU 25 µM was selected for downstream validation. This dose pair showed consistent activity in HCT116 spheroids and RKO cell aggregates and was used for follow-up imaging and Western blot analysis.

### 2.6. Selected Sulfasalazine and 5-Fluorouracil Treatment Combinations Alter the Growth of Established Spheroids

**Figure 6:**
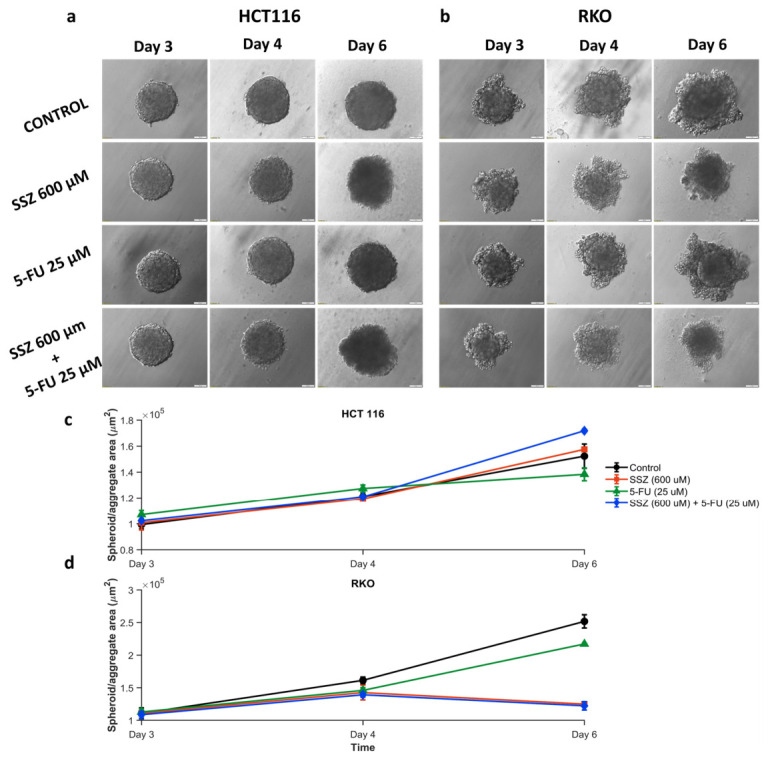
Combined sulfasalazine and 5-FU treatment modulates the growth of established CRC spheroids/aggregates. (**a**,**b**) Representative bright-field images of HCT116 spheroids and RKO cell aggregates treated with DMSO control, sulfasalazine (SSZ, 600 µM), 5-fluorouracil (5-FU, 25 µM), or combined SSZ + 5-FU. Spheroids/aggregates were first allowed to form for 3 days before treatment initiation. SSZ treatment was applied on Day 3, followed by 5-FU treatment on Day 4, and spheroids/aggregates were monitored through Day 6. (**c**,**d**) Quantitative analysis of spheroid/aggregate area over time in HCT116 and RKO models. Spheroid/aggregate area was measured from calibrated bright-field images using ImageJ/Fiji version 2.18.0. In contrast to RKO cells, the SSZ + 5-FU combination did not reduce spheroid area in HCT116 cells. Instead, treated spheroids exhibited a less compact structure and less clearly defined borders, suggesting an effect on spheroid organization. In contrast, RKO aggregates showed a stronger treatment response, with SSZ-containing treatments limiting aggregate expansion, particularly by Day 6. Data are presented as mean ± SD. Statistical significance was determined by two-way ANOVA followed by post hoc comparisons versus the day-matched control group, with *p* < 0.05 considered statistically significant. Scale bars = 100 µm.

To understand the effect of the selected treatment condition on established 3D CRC cultures, treatment with SSZ (600 µM) and 5-FU (25 µM), either alone or in combination, was applied. Spheroids/aggregates were monitored by bright-field imaging from Day 3 to Day 6 (Figure 6a,b). In the HCT116 model, control spheroids continued to grow during this period and maintained a rounded morphology (Figure 6a). The SSZ + 5-FU combination group showed visible morphological changes and disruption in spheroid borders; however, quantitative analysis of spheroid/aggregate area did not show a reduction compared with the control group by Day 6 (Figure 6c). In contrast, RKO cultures showed a stronger treatment response based on spheroid/aggregate area (Figure 6b,d). Control RKO aggregates increased markedly over time, whereas SSZ-containing treatments limited aggregate expansion. The strongest effect was observed in the SSZ + 5-FU group, which showed smaller aggregates on Day 6 relative to the control group (Figure 6d). Overall, time-course quantification supported a stronger inhibitory response in RKO aggregates than in HCT116 spheroids, particularly in the SSZ-containing groups.

### 2.7. Combined Sulfasalazine and 5-Fluorouracil Treatment Suppresses KRAS/MAPK Signaling in CRC Spheroids Formed in PEGDA Microwells

**Figure 7:**
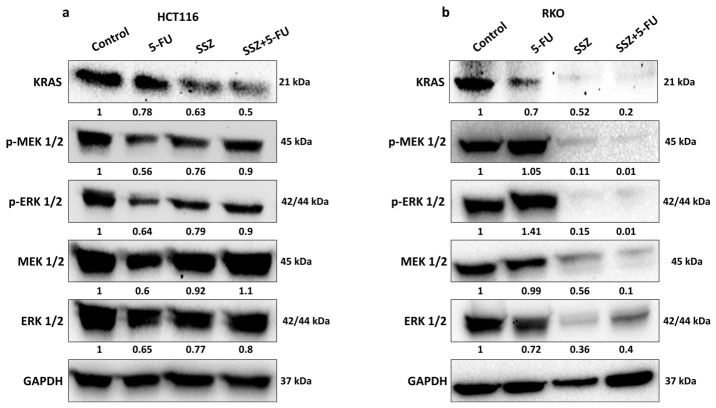
(**a**,**b**) Sulfasalazine and 5-FU modulate KRAS/MAPK pathway proteins in PEGDA microwell-derived CRC spheroids. Representative Western blot analysis of KRAS, p-MEK1/2, total MEK1/2, p-ERK1/2, and total ERK1/2 in HCT116 spheroids and RKO cell aggregates after treatment with control, 5-FU, sulfasalazine (SSZ), or SSZ + 5-FU. GAPDH was used as the loading control. Densitometric values shown below each band were calculated by first normalizing each protein band intensity to GAPDH, followed by scaling to the untreated control group, which was set to 1.00. SSZ + 5-FU reduced KRAS expression in both HCT116 and RKO models. In RKO cell aggregates, treatments with SSZ reduced p-MEK1/2 and p-ERK1/2 levels, with the combination group showing the largest decrease. KRAS expression was decreased post-treatment in HCT116 spheroids, but to a lesser extent than observed in RKO for downstream MAPK inhibition.

Western blot analysis was performed on PEGDA microwell-derived 3D cultures to assess whether sulfasalazine (SSZ, 600 µM) and 5-FU (25 µM) altered KRAS/MAPK-associated signaling after treatment of established spheroids. The panel included KRAS, p-MEK1/2, total MEK1/2, p-ERK1/2, total ERK1/2, and GAPDH as the loading control (Figure 7). Values shown below the bands represent GAPDH-normalized fold changes relative to the untreated control. In parallel, p-MEK1/2/total MEK1/2 and p-ERK1/2/total ERK1/2 ratios were calculated to evaluate pathway activation. In HCT116 spheroids, KRAS expression decreased after treatment, with the strongest reduction observed in the SSZ + 5-FU group. GAPDH-normalized p-MEK1/2 and p-ERK1/2 levels were also reduced compared with the control. However, when phosphorylation was evaluated relative to total MEK1/2 and total ERK1/2, the downstream MAPK response was more modest, particularly for ERK. This suggests that SSZ-containing treatment reduced KRAS expression in HCT116 spheroids but did not strongly suppress downstream ERK activation under these conditions. In RKO cell aggregates, SSZ-containing treatments produced a stronger pathway response. KRAS, p-MEK1/2, and p-ERK1/2 levels were reduced, and the p-MEK1/2/total MEK1/2 and p-ERK1/2/total ERK1/2 ratios showed clearer pathway suppression, especially in the combination group. These results indicate that SSZ + 5-FU modulated KRAS/MAPK-associated signaling in PEGDA microwell-derived CRC spheroids/aggregates, with a stronger downstream inhibitory response in RKO compared with HCT116.

### 2.8. Combined Sulfasalazine and 5-Fluorouracil Treatment Regulates PARP Cleavage-Associated Apoptotic Signaling, GPX-4-Dependent Redox Defense, Proliferation, and Stemness-Associated Markers in PEGDA Microwell-Derived CRC Spheroids

**Figure 8:**
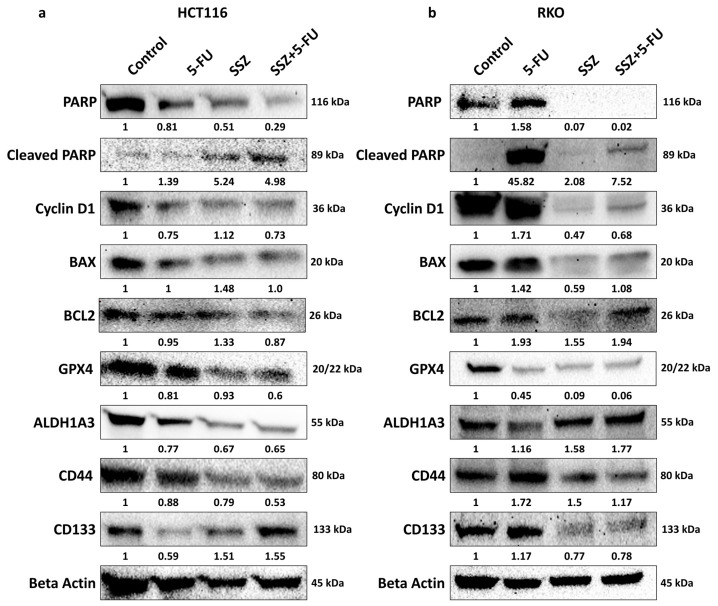
(**a**,**b**) Sulfasalazine and 5-FU modulate apoptosis-, redox defense-, proliferation-, and stemness-associated proteins in PEGDA microwell-derived CRC spheroids. Representative Western blot analysis of PARP, cleaved PARP, Cyclin D1, Bax, Bcl-2, GPX4, ALDH1A3, CD44, and CD133 in HCT116 spheroids and RKO cell aggregates after treatment with control, 5-FU, sulfasalazine (SSZ), or SSZ + 5-FU. β-actin was used as the loading control. Densitometric values shown below each band were calculated by first normalizing each target protein band intensity to β-actin, followed by scaling to the untreated control group, which was set to 1.00. SSZ + 5-FU increased apoptosis-associated PARP cleavage in HCT116 spheroids. In RKO cell aggregates, interpretation of the cleaved PARP/PARP ratio needs caution because the increase was partly influenced by the strong reduction in full-length PARP. SSZ-containing treatments also downregulated GPX4 and Cyclin D1 in a cell-line-dependent manner, suggesting modulation of redox defense-associated and proliferative signaling. Treatment- and cell-line-specific changes were observed for ALDH1A3, CD44, and CD133, which were indicative of a modulation and not a complete suppression of stemness-associated markers.

Additional Western blot analysis was performed to examine whether SSZ and 5-FU altered proteins associated with apoptosis, proliferation, redox defense, and stemness-associated phenotypes in PEGDA microwell-derived 3D cultures (Figure 8). The panel consisted of PARP, cleaved PARP, Cyclin D1, Bax, Bcl-2, GPX4, ALDH1A3, CD44, CD133, and β-actin as a loading control. Values below the bands are β-actin-normalized fold changes compared to the untreated control. Apoptosis-associated signaling was evaluated by determining cleaved PARP/PARP and Bax/Bcl-2 ratios from normalized band intensities. In HCT116 spheroids, treatment reduced full-length PARP and increased cleaved PARP, with the cleaved PARP/PARP ratio showing the strongest increase in the SSZ + 5-FU group. This supports PARP cleavage-associated apoptotic signaling in HCT116 spheroids. Bax/Bcl-2 changed only modestly, so the apoptosis-associated interpretation was based mainly on PARP processing rather than a strong Bax/Bcl-2 shift. GPX4 showed the clearest reduction in the combination group, suggesting altered GPX4-associated redox defense capacity. ALDH1A3 and CD44 levels decreased after treatment, particularly in the combination group, whereas CD133 showed a variable response. In RKO cell aggregates, SSZ-containing treatments reduced GPX4, suggesting modulation of redox defense capacity. Moreover, SSZ-containing treatments also reduced Cyclin D1 proliferative signaling. PARP was markedly reduced after SSZ and SSZ + 5-FU treatment. Although the derived cleaved PARP/PARP ratio increased strongly, this increase was partly driven by the near-complete loss of full-length PARP in SSZ-containing groups. Therefore, apoptosis-associated signaling in RKO should be interpreted cautiously and not solely from the cleaved PARP/PARP ratio. Bax/Bcl-2 did not consistently increase, suggesting that the RKO response was not primarily associated with a Bax/Bcl-2 shift under these conditions. CD133 decreased after SSZ-containing treatment, while ALDH1A3 and CD44 showed more variable responses. Together, these results indicate that SSZ + 5-FU modulated apoptosis-associated PARP processing, GPX4-associated redox defense, Cyclin D1-associated proliferation, and stemness-associated markers in a cell-line-dependent manner. Importantly, here, we interpret changes in GPX4 and ALDH1A3 as redox defense- and therapy-resistance-associated marker changes, but not as definite evidence of ferroptosis.

## 3. Discussion

Treatment failure in CRC often occurs when tumors do not fully respond to fluoropyrimidine-based chemotherapy or when drug-resistant tumor cell populations emerge. Although 5-FU remains a key part of CRC treatment, resistance can develop through changes in drug metabolism, DNA-damage response, apoptosis escape, redox adaptation, and stem-like cell states [4,5,32,33,34]. For this reason, it is important to test treatment approaches that target survival pathways beyond DNA synthesis. Previously, adaptive stress-survival pathways have been investigated in CRC, with reported roles in tumor-cell survival, proliferation, invasion, tumor growth, stemness-associated behavior, and treatment response [35,36,37,38].

In our study, *KRAS* signaling was selected as a strategic target due to its frequent alterations in CRC and its regulatory role in the RAF/MEK/ERK pathway. Our public dataset analysis (cBioPortal) of a combined colorectal cancer cohort from three studies, including 1228 samples from 1225 patients, showed *KRAS* alterations in 473 patients (39% of queried patients). Mutation analysis showed a somatic *KRAS* mutation frequency of 37.8%, with most alterations classified as missense mutations and concentrated in canonical hotspot regions. The Kaplan–Meier Plotter analysis using the Affymetrix probe (214352_s_at) demonstrated that elevated KRAS expression was associated with a shorter OS in 1061 CRC patients. The high-expression cohort had a median overall survival of 98 months, compared with 137 months in the low-expression cohort, with an HR of 1.34 and a log-rank *p*-value of 0.0065. DepMap CRISPR Gene Dependency analysis further showed enriched *KRAS* dependency patterns in 96 colorectal adenocarcinoma models, including COAD colon adenocarcinoma models and READ rectal adenocarcinoma models. Thus, these public dataset findings show *KRAS*-associated signaling as an important molecular target in CRC and support evaluation of this pathway in the SSZ/5-FU treatment response.

SSZ inhibits system x_c−, limiting cystine import and glutathione synthesis [14,15,16]. Prior work by Ma et al. showed that SSZ can sensitize CRC cells to cisplatin via a glutathione-dependent mechanism [16]. In our study, SSZ alone decreased the viability and colony formation of CRC cells in 2D cultures. SSZ in combination with 5-FU also decreased early spheroid formation and potentiated the inhibitory effect of 5-FU on early 3D spheroid/aggregate viability. In established spheroids, SSZ-containing treatments affected spheroid/aggregate growth, with a profound inhibitory effect on RKO aggregates. In a similar vein, Kerkhove et al. showed the potential of SSZ to sensitize CRC to treatment under hypoxia, supporting the concept of targeting cystine/glutathione metabolism to increase sensitivity to radiotherapy in 3D CRC models [39].

Spheroids capture features that are not fully represented in monolayer culture, including cell–cell contact, compact architecture, and diffusion-limited drug exposure [18,19,20,21]. By separating early spheroid formation from treatment of established spheroids, this study evaluated whether SSZ affects both initial 3D spheroid/aggregate establishment and treatment response after 3D organization has already occurred. This distinction is important because a drug may interfere with early aggregate formation but show weaker activity once the spheroid is already established. In our models, treatments with SSZ exerted clear effects in early spheroid formation. In established spheroids, the response was more cell-line-dependent, with RKO aggregates showing stronger inhibition of aggregate expansion than HCT116 spheroids. These findings support the value of evaluating drug response in both early-forming and established 3D CRC models. This study also builds on our previous CRC work using the PEGDA-based microwell platform. In that study, we established a 3D CRC spheroid model in PEGDA microwells and showed that miR-873 suppressed *KRAS*-associated signaling, reducing CRC proliferation, colony formation, invasion, and 3D spheroid growth while improving 5-FU response [23].

Building on our previous *KRAS*-focused CRC work, this study tested SSZ as a clinically approved, repurposed agent that may offer a more pharmacologically practical approach for improving 5-FU response in CRC models. Western blot results supported treatment-associated modulation of KRAS/MAPK-associated signaling in PEGDA microwell-derived spheroids. SSZ treatments decreased KRAS expression in both HCT116 and RKO models, but downstream pathway responses were different in the two cell lines. RKO exhibited more prominent decreases in p-MEK/total MEK and p-ERK/total ERK, especially in the SSZ + 5-FU group. KRAS was downregulated in HCT116, but p-ERK/total ERK was relatively stable, indicating weaker downstream MAPK suppression at the examined time point. This difference may reflect the distinct molecular backgrounds of the models, including KRAS-mutant HCT116 and KRAS-wild-type RKO. These findings are consistent with prior CRC work reporting that SSZ reduced cell viability, colony formation, sphere formation, ALDH activity, and KRAS/MMP7/CD44 expression in CRC cell lines, supporting the relevance of SSZ to growth- and stemness-associated signaling in CRC [17]. Therefore, our data suggest that SSZ/5-FU can modulate KRAS-associated signaling in 3D CRC spheroids, although the magnitude of downstream MAPK suppression may depend on the cell-line context.

PARP cleavage is widely recognized as an apoptosis-associated marker and reflects caspase-mediated proteolytic processing during apoptotic cell death [40]. Thus, cleaved PARP/PARP was used as the main apoptosis-related ratio. The SSZ + 5-FU group showed the maximal increase in cleaved PARP/PARP in HCT116 spheroids, which supports combination-associated apoptotic signaling. Bax/Bcl-2 was also calculated to assess the balance between pro-apoptotic and anti-apoptotic signaling; however, this ratio changed modestly and was not consistent across the two cell lines. Therefore, apoptotic signaling was mainly supported by PARP rather than a robust Bax/Bcl-2-mediated mechanism. In RKO aggregates, the cleaved PARP/PARP ratio increased strongly in SSZ-containing groups, but this result should be interpreted cautiously because full-length PARP was markedly reduced, which can mathematically increase the ratio. Therefore, further analyses of flow cytometry-based Annexin V/I assay and caspase activity assessments are needed to demonstrate direct regulation of apoptotic cell death mechanisms. Moreover, cystine uptake, glutathione metabolism and spheroid growth are closely linked to redox adaptation and treatment resistance; we also examined GPX4 and markers related to therapy resistance and stemness. The decrease in GPX4 suggests that the cells may have weaker GPX4-related redox defense capacity. These results are in line with previous CRC studies showing that SSZ can disrupt xCT-dependent redox homeostasis and increase sensitivity to treatment under hypoxic conditions [39]. However, we believe that GPX-4 reduction alone may not be sufficient to understand ferroptosis. Direct assays for glutathione depletion, reactive oxygen species accumulation, lipid peroxidation, iron dependence, and ferroptosis rescue would be useful to confirm ferroptosis involvement [41,42,43].

Our data also suggests that treatment with SSZ may affect proteins related to stemness and therapy resistance in spheroids, as shown by the changes in ALDH1A3, CD44, and CD133. This idea is further supported by the reduction in the size of early spheroids.

### Limitations and Future Studies

Overall, our results support sulfasalazine (SSZ) as a potential repurposed therapeutic partner for 5-fluorouracil (5-FU) in in vitro colorectal cancer (CRC) models. Further studies are needed to elucidate the underlying mechanisms of cell proliferation and cell death mechanisms after combined treatment. In particular, investigations that incorporate apoptosis, redox and ferroptosis analyses, functional stemness assays, in vivo CRC models and complementary computational modeling could provide deeper insights into the molecular pathways and biological processes responsible for the observed therapeutic effects. Moreover, further three-dimensional characterization by Z-stack imaging on spheroids may give detailed insights into the observed morphological changes. We plan to design new experimental protocols incorporating flow cytometry-based Annexin V apoptosis detection, redox and ferroptosis assays, functional stemness assays and in vivo CRC studies. These investigations are expected to provide deeper insights into the underlying mechanisms and translational potential of this combination therapy for CRC treatment.

## 4. Materials and Methods

### 4.1. Public Dataset Evaluation

Publicly available cBioPortal (https://www.cbioportal.org/, accessed on 8 April 2026), Kaplan–Meier Plotter (https://kmplot.com/, accessed on 8 April 2026), and DepMap (https://depmap.org/portal/, accessed on 8 April 2026), resources were used for public dataset evaluation. cBioPortal was used to analyze colorectal cancer cohort data for *KRAS* mutation frequency and *KRAS* mRNA expression [27,28]. The colorectal cancer cohort included 1228 samples for mRNA expression analysis and 1225 samples for mutation analysis. Kaplan–Meier Plotter was used to evaluate the association between KRAS expression and overall survival in colorectal cancer using the *KRAS* Affymetrix probe 214352_s_at in 1061 patients [29]. DepMap CRISPR Gene Dependency data were used to assess *KRAS* dependency enrichment across colorectal adenocarcinoma models [30]. The colorectal adenocarcinoma dataset included 96 models, including the COAD colon adenocarcinoma and READ rectal adenocarcinoma model subgroups.

### 4.2. Cell Lines and Culture

The human colorectal cancer cell lines used in this study were HCT116 and RKO. HCT116 cells (*KRAS* G13D) and RKO cells (*KRAS* wild type) were purchased from the American Type Culture Collection (ATCC, Manassas, VA, USA). HCT116 cells were cultured in McCoy’s 5A medium (ATCC), and RKO cells were cultured in Eagle’s Minimum Essential Medium (EMEM; ATCC). Both media were supplemented with 10% fetal bovine serum (FBS; Thermo Fisher Scientific, Waltham, MA, USA) and 1% penicillin–streptomycin (Thermo Fisher Scientific). Cells were grown in a humidified incubator at 37 °C and 5% CO_2_ and regularly passaged before confluency.

### 4.3. Reagents and Drug Preparation

Sulfasalazine (SSZ; Sigma-Aldrich, St. Louis, MO, USA; S0883) was dissolved in dimethyl sulfoxide (DMSO; Santa Cruz Biotechnology, Dallas, TX, USA) as a stock solution. 5-fluorouracil (5-FU; Sigma-Aldrich) was also prepared in DMSO and diluted freshly in complete growth medium before treatment. DMSO-treated cultures were used as solvent controls where applicable, and the final DMSO concentration was kept consistent in drug-treated and control groups. For the early-spheroid-formation assay, SSZ was used at 300 µM based on the 2D dose–response results, and 5-FU was used at 25 µM as the selected combination-treatment dose. For established spheroid dose-matrix studies, SSZ was tested at 0, 200, 300, 400, 600, 800, 1200, and 1600 µM, and 5-FU was tested at 0, 12.5, 25, and 50 µM. For downstream imaging validation and Western blot experiments, SSZ 600 µM and 5-FU 25 µM were used unless otherwise indicated.

### 4.4. Sulfasalazine Dose–Response MTS Assay

For 2D dose–response analysis, HCT116 and RKO cells were seeded at 1500 cells/well in 96-well plates and allowed to adhere. Cells were seeded six hours before treatment with increasing concentrations of SSZ and incubated for 72 h. Cell viability was measured using the CellTiter 96 AQueous One Solution MTS assay (Promega, Madison, WI, USA) according to the manufacturer’s instructions and a previously described MTS-based viability assay [44]. Absorbance was measured at 490 nm using an Epoch microplate spectrophotometer (Agilent BioTek, Winooski, VT, USA) with Gen5 software version 1.09 (Agilent BioTek, Winooski, VT, USA). Values were normalized to the matched DMSO control group (set at 100%).

### 4.5. Colony Formation Assay

For colony formation assays, HCT116 and RKO cells were seeded at 500 cells per well in 6-well plates as previously described [45]. One day after seeding, cells were treated with the indicated concentrations of SSZ and then allowed to grow until visible colonies formed in the control wells. Colonies were fixed and stained with crystal violet solution (Sigma-Aldrich), imaged under matched conditions, and quantified using ImageJ/Fiji software version 2.18.0 (National Institutes of Health, Bethesda, MD, USA). Colony number was normalized to the matched control group and expressed as a percentage of relative colony formation.

### 4.6. Early-Spheroid-Formation Assay in Ultra-Low-Attachment Plates

To assess whether SSZ interferes with the initial formation of CRC spheroids, the early-spheroid-formation assay was developed. HCT116 and RKO cells were seeded at 1500 cells/well in ultra-low-attachment U-bottom 96-well Nunclon Sphera plates (Thermo Fisher Scientific, Waltham, MA, USA). On Day 0, 6 h after seeding, SSZ was added to the assigned wells. On Day 1, 5-FU was added to the respective treatment groups. Control, SSZ-alone, 5-FU-alone, and SSZ plus 5-FU treatment groups were established. Spheroids were imaged on Day 3 using an Olympus IX73 inverted microscope (Olympus Corporation, Tokyo, Japan) under matched bright-field imaging conditions. Spheroid/aggregate area was measured by ImageJ/Fiji. Metabolic viability was assessed using the PrestoBlue HS Cell Viability Reagent (Thermo Fisher Scientific). Reagents were incubated at 37 °C, and Fluorescence was measured using a BioTek Synergy H1 multimode plate reader (Agilent BioTek) with Gen5 software version 1.11 (Agilent BioTek). Fluorescence values were background-corrected and normalized to the control group.

### 4.7. Combination Dose-Matrix Assay, Imaging Validation, and SynergyFinder+ Analysis in Established Spheroids

To evaluate drug response in already-formed spheroids, HCT116 and RKO cells were seeded at a density of 1500 cells per well in ultra-low-attachment U-bottom 96-well plates and cultured for 3 days to allow spheroid establishment. On Day 3, spheroids were treated with SSZ at 0, 200, 300, 400, 600, 800, 1200, or 1600 µM. On Day 4, 5-FU was added to the respective matrix wells at concentrations of 0, 12.5, 25, and 50 µM. Metabolic viability was determined on Day 6 via the PrestoBlue HS reagent after incubation for 3 h at 37 °C. Fluorescence was measured using the BioTek Synergy H1 multimode plate reader (Agilent BioTek) and normalized to the DMSO control group. Drug-interaction patterns were analyzed with the zero interaction potency (ZIP), highest single agent (HSA), Bliss, and Loewe models using SynergyFinder+ (https://synergyfinder.fimm.fi/, accessed on 13 March 2026). Dose pairs were selected based on dose-matrix analysis and were validated by bright-field imaging before treatment and at follow-up on Day 6 for spheroid/aggregate size and morphology. To quantify the established spheroids/aggregates shown in Figure 6, spheroid/aggregate area was measured from calibrated bright-field images using ImageJ/Fiji. Measurements were performed on three independent spheroids/aggregates per condition at each time point. Area values were reported in µm^2^ and plotted over time as mean ± SD.

### 4.8. PEGDA Microwell Spheroid Generation

PEGDA microwell-derived spheroids were generated using our previously established 3D CRC microwell platform [46]. Briefly, glass coverslips were functionalized with 3-(trimethoxysilyl)propyl methacrylate (TMSPMA; Sigma-Aldrich), and microwell arrays were fabricated using 40% (*w*/*v*) PEGDA(MW 700; Sigma-Aldrich) in phosphate-buffered saline (PBS; Sigma-Aldrich) containing 0.2% (*w*/*v*) photoinitiator (2-hydroxy-4′-(2-hydroxyethoxy)-2-methylpropiophenone; Sigma-Aldrich). Polymerization was performed using an OmniCure Series 2000 UV spot curing system (Lumen Dynamics Group Inc., Mississauga, ON, Canada) and a chrome photomask containing circular microwell features. Fabricated chips were rinsed, equilibrated in sterile PBS or complete medium, and seeded with approximately 0.8 × 10^6^ HCT116 or RKO cells per chip. Chips were incubated at 37 °C with 5% CO_2_, and spheroids were allowed to form for 3 days before treatment.

### 4.9. Treatment and Sampling of PEGDA Microwell Spheroids

The established spheroid treatment schedule was used to treat the PEGDA microwell spheroids. Formation of spheroids was allowed for 3 days before exposure to drugs. SSZ was added to the defined treatment groups at Day 3. On the 4th day, 5-FU was added to each group. On Day 6, spheroids were collected for protein extraction and Western blot analysis. This design allowed molecular pathway analysis after treatment of already-formed 3D CRC spheroids.

### 4.10. Western Blot Analysis

For 2D Western blot analysis shown in Figure 2, HCT116 and RKO cells were treated with SSZ under the same dose–response conditions used for the 2D viability assay, collected after 72 h, and processed for protein extraction as described below. For PEGDA microwell-derived 3D cultures, spheroids were collected on Day 6 and lysed in ice-cold protein extraction buffer supplemented with protease and phosphatase inhibitors, as previously described [47]. Protein concentration was measured using a Pierce BCA Protein Assay Kit (Thermo Fisher Scientific). Equal amounts of protein were mixed with Laemmli sample buffer (Bio-Rad, Hercules, CA, USA), resolved using 12% Mini-PROTEAN TGX precast gels (Bio-Rad), and transferred to PVDF membranes (Thermo Fisher Scientific). Membranes were blocked in 5% nonfat milk in TBS-T and incubated overnight at 4 °C with primary antibodies against KRAS, p-MEK1/2, MEK1/2, p-ERK1/2, ERK1/2, PARP, cleaved PARP, Cyclin D1, Bax, Bcl-2, GPX4, ALDH1A3, CD44, CD133, GAPDH, and β-actin. KRAS antibody was obtained from Santa Cruz Biotechnology (Dallas, TX, USA), ALDH1A3 antibody was obtained from Abcam (Anti-ALDH1A3 antibody, ab129815; Cambridge, UK), and MAPK, apoptosis, proliferation, stemness-associated, redox defense, and loading-control antibodies were obtained from Cell Signaling Technology (Danvers, MA, USA), unless otherwise indicated. Membranes were incubated with horseradish peroxidase (HRP)-conjugated secondary antibodies, developed using SignalFire Plus enhanced chemiluminescence (ECL) Reagent (Cell Signaling Technology), and imaged using a ChemiDoc XRS+ imaging system (Bio-Rad). Densitometry was performed using Image Lab software version 4.1 (Bio-Rad). For the densitometric values shown below the Western blot bands, raw integrated band intensities were first normalized to the corresponding loading control, GAPDH for Figure 7 and β-actin for Figure 8. Each normalized value was then divided by the untreated control value, thereby setting the control group to 1.00. For pathway-specific interpretation, p-MEK1/2/total MEK1/2 and p-ERK1/2/total ERK1/2 ratios were additionally calculated from normalized band intensities. For apoptosis-associated interpretation, cleaved PARP/PARP and Bax/Bcl-2 ratios were calculated from normalized band intensities and scaled to the untreated control group.

### 4.11. Statistical Analysis

Data are presented as mean ± standard deviation (SD) unless otherwise stated. For normalized assays, the control mean was set to 100%, while individual control replicate values were retained to reflect biological or technical variation. Two-group comparisons were performed using unpaired, two-tailed Student’s t-tests when appropriate. Multiple-group comparisons were performed using one-way ANOVA followed by appropriate post hoc testing, including Dunnett’s test for comparisons against the control group. For time-course spheroid/aggregate area analyses, two-way ANOVA was used with treatment group and time point as the two factors, followed by post hoc comparisons between each treatment group and the corresponding day-matched control group. A *p*-value below 0.05 was considered statistically significant. Drug-combination responses were analyzed using SynergyFinder+ with the ZIP, HSA, Bliss, and Loewe models. Graphing and statistical analysis were performed using GraphPad Prism version 11.0.2 (GraphPad Software, Boston, MA, USA), and MATLAB version R2026a (MathWorks, Natick, MA, USA) was used for time-course plot generation.

## 5. Conclusions

In this work, we showed that sulfasalazine suppressed CRC cell growth as a single agent and improved the response to 5-FU in 3D spheroid models. SSZ reduced cell viability and colony formation in 2D cultures and was associated with decreased KRAS expression in both HCT116 and RKO cells. In 3D cultures, SSZ reduced early spheroid formation. SSZ-containing treatments changed the spheroid/aggregate growth in established spheroid cultures. RKO aggregates showed a stronger inhibitory response than HCT116 spheroids. The selected SSZ + 5-FU treatment also affected several molecular markers linked to treatment response. In PEGDA microwell-derived spheroids, this combination reduced KRAS expression, suppressed KRAS/MAPK-related signaling, increased PARP cleavage, lowered GPX4 expression, and altered Cyclin D1 and stemness-related markers. Together, these findings suggest that SSZ may improve the response to 5-FU in CRC spheroid models by acting on several survival-related pathways. This work also supports the use of our PEGDA microwell-based 3D platform as a practical model for testing drug combinations in CRC. Since *KRAS*-associated signaling and therapy resistance remain major problems in CRC treatment, this strategy may be useful for studying cell-line- and context-dependent treatment responses, including in *KRAS*-mutant or *KRAS*-dependent tumors.

Although our study showed that SSZ significantly enhanced the response to 5-FU in CRC models with different *KRAS* backgrounds, further studies are needed to elucidate the underlying mechanisms of the combined treatment. In particular, investigations that incorporate Annexin V/I-based apoptosis detection, redox and ferroptosis analyses, functional stemness assays, in vivo CRC models and complementary computational modeling could provide deeper insights into the molecular pathways and biological processes responsible for the observed therapeutic effects.

## Data Availability

The datasets generated and/or analyzed during the current study are available from the corresponding author on reasonable request. Publicly available data analyzed in this study were obtained from cBioPortal, Kaplan–Meier Plotter, DepMap, and SynergyFinder+, and are accessible through their respective online platforms.

## Abbreviations

The following abbreviations are used in this manuscript:

CRC	Colorectal Cancer
5-FU	5-Fluorouracil
FDA	U.S. Food and Drug Administration
SSZ	Sulfasalazine
*KRAS*	Kirsten rat sarcoma viral oncogene homolog
MAPK	Mitogen-activated protein kinase
PEGDA	Poly(ethylene glycol) diacrylate
2D	Two-dimensional
3D	Three-dimensional
COAD	Colon adenocarcinoma
READ	Rectal adenocarcinoma
DMSO	Dimethyl sulfoxide
MTS	3-(4,5-dimethylthiazol-2-yl)-5-(3-carboxymethoxyphenyl) -2-(4-sulfophenyl)-2H-tetrazolium
GPX4	Glutathione peroxidase 4
ALDH1A3	Aldehyde dehydrogenase 1 family member A3
PARP	Poly(ADP-ribose) polymerase
MEK	Mitogen-activated protein kinase kinase
ERK	Extracellular signal-regulated kinase
BCA	Bicinchoninic acid
PVDF	Polyvinylidene difluoride
HRP	Horseradish peroxidase
ECL	Enhanced chemiluminescence
HR	Hazard ratio
SD	Standard deviation
ZIP	Zero interaction potency
HSA	Highest single agent

## References

[1] Wu S., Zhang Y., Lin Z., Wei M. (2025). Global Burden of Colorectal Cancer in 2022 and Projections to 2050: Incidence and Mortality Estimates from GLOBOCAN. BMC Cancer.

[2] Eng C., Yoshino T., Ruíz-García E., Mostafa N., Cann C.G., O’Brian B., Benny A., Perez R.O., Cremolini C. (2024). Colorectal Cancer. Lancet.

[3] Siegel R.L., Wagle N.S., Star J., Kratzer T.B., Smith R.A., Jemal A. (2026). Colorectal Cancer Statistics, 2026. CA Cancer J. Clin..

[4] Vodenkova S., Buchler T., Cervena K., Veskrnova V., Vodicka P., Vymetalkova V. (2020). 5-Fluorouracil and Other Fluoropyrimidines in Colorectal Cancer: Past, Present and Future. Pharmacol. Ther..

[5] Gmeiner W.H., Okechukwu C.C. (2023). Review of 5-FU Resistance Mechanisms in Colorectal Cancer: Clinical Significance of Attenuated on-Target Effects. Cancer Drug Resist..

[6] Takeda M., Yoshida S., Inoue T., Sekido Y., Hata T., Hamabe A., Ogino T., Miyoshi N., Uemura M., Yamamoto H. (2025). The Role of KRAS Mutations in Colorectal Cancer: Biological Insights, Clinical Implications, and Future Therapeutic Perspectives. Cancers.

[7] Huang L., Guo Z., Wang F., Fu L. (2021). KRAS Mutation: From Undruggable to Druggable in Cancer. Signal Transduct. Target. Ther..

[8] Hu S.-S., Han Y., Tan T.-Y., Chen H., Gao J.-W., Wang L., Yang M.-H., Zhao L., Wang Y.-Q., Ding Y.-Q. (2023). SLC25A21 Downregulation Promotes KRAS-Mutant Colorectal Cancer Progression by Increasing Glutamine Anaplerosis. JCI Insight.

[9] Sethy C., Kundu C.N. (2021). 5-Fluorouracil (5-FU) Resistance and the New Strategy to Enhance the Sensitivity against Cancer: Implication of DNA Repair Inhibition. Biomed. Pharmacother..

[10] Srinivas U.S., Dyczkowski J., Beißbarth T., Gaedcke J., Mansour W.Y., Borgmann K., Dobbelstein M. (2015). 5-Fluorouracil Sensitizes Colorectal Tumor Cells towards Double Stranded DNA Breaks by Interfering with Homologous Recombination Repair. Oncotarget.

[11] Meng M., Zhong K., Jiang T., Liu Z., Kwan H.Y., Su T. (2021). The Current Understanding on the Impact of KRAS on Colorectal Cancer. Biomed. Pharmacother..

[12] Rahman S., Garrel S., Gerber M., Maitra R., Goel S. (2021). Therapeutic Targets of KRAS in Colorectal Cancer. Cancers.

[13] Plosker G.L., Croom K.F. (2005). Sulfasalazine: A Review of Its Use in the Management of Rheumatoid Arthritis. Drugs.

[14] Choi J., Patel P., Fenando A. (2026). Sulfasalazine. StatPearls.

[15] Lin W., Wang C., Liu G., Bi C., Wang X., Zhou Q., Jin H. (2020). SLC7A11/xCT in Cancer: Biological Functions and Therapeutic Implications. Am. J. Cancer Res..

[16] Ma M., Chen G., Wang P., Lu W., Zhu C., Song M., Yang J., Wen S., Xu R., Hu Y. (2015). Xc−Inhibitor Sulfasalazine Sensitizes Colorectal Cancer to Cisplatin by a GSH-Dependent Mechanism. Cancer Lett..

[17] Leung W.-H., Shih J.-W., Chen J.-S., Mokgautsi N., Wei P.-L., Huang Y.-J. (2022). Preclinical Identification of Sulfasalazine’s Therapeutic Potential for Suppressing Colorectal Cancer Stemness and Metastasis through Targeting KRAS/MMP7/CD44 Signaling. Biomedicines.

[18] Yau J.N.N., Adriani G. (2023). Three-Dimensional Heterotypic Colorectal Cancer Spheroid Models for Evaluation of Drug Response. Front. Oncol..

[19] Ahmad Zawawi S.S., Salleh E.A., Musa M. (2024). Spheroids and Organoids Derived from Colorectal Cancer as Tools for in Vitro Drug Screening. Explor. Target. Anti-Tumor Ther..

[20] Arora S., Singh S., Mittal A., Desai N., Khatri D.K., Gugulothu D., Lather V., Pandita D., Vora L.K. (2024). Spheroids in Cancer Research: Recent Advances and Opportunities. J. Drug Deliv. Sci. Technol..

[21] Lamichhane A., Tavana H. (2024). Three-Dimensional Tumor Models to Study Cancer Stemness-Mediated Drug Resistance. Cell. Mol. Bioeng..

[22] Rafiei S., Ghanbari-Abdolmaleki M., Zeinali R., Heidari-Keshel S., Rahimi A., Royanian F., Zaeifi D., Taheri K., Pourtaghi K., Khaleghi M. (2024). Silk Fibroin/Vitreous Humor Hydrogel Scaffold Modified by a Carbodiimide Crosslinker for Wound Healing. Biopolymers.

[23] Bandegi M., Biltekin E., Akay Y.M., Ozpolat B., Akay M. (2026). MicroRNA-873 Suppresses Viability and Invasion of Colorectal Cancer Through KRAS/MAPK Signaling and Sensitizes Tumor Spheroids to 5-Fluorouracil in a 3D Microwell Model. IEEE Open J. Eng. Med. Biol..

[24] Mendieta M., Hatami M., Singh M., Fard S.S., Dehshiri M., Schill A., Nevozhay D., Aglyamov S., Ozpolat B., Sokolov K.V. (2025). Non-Invasive Measurement of Elasticity in Glioblastoma Multiforme Validates Decreased TMZ Sensitivity in Astrocyte Co-Culture. IEEE Open J. Eng. Med. Biol..

[25] Zheng S., Wang W., Aldahdooh J., Malyutina A., Shadbahr T., Tanoli Z., Pessia A., Tang J. (2022). SynergyFinder Plus: Toward Better Interpretation and Annotation of Drug Combination Screening Datasets. Genom. Proteom. Bioinform..

[26] Ianevski A., He L., Aittokallio T., Tang J. (2017). SynergyFinder: A Web Application for Analyzing Drug Combination Dose–Response Matrix Data. Bioinformatics.

[27] Gao J., Aksoy B.A., Dogrusoz U., Dresdner G., Gross B., Sumer S.O., Sun Y., Jacobsen A., Sinha R., Larsson E. (2013). Integrative Analysis of Complex Cancer Genomics and Clinical Profiles Using the cBioPortal. Sci. Signal..

[28] De Bruijn I., Kundra R., Mastrogiacomo B., Tran T.N., Sikina L., Mazor T., Li X., Ochoa A., Zhao G., Lai B. (2023). Analysis and Visualization of Longitudinal Genomic and Clinical Data from the AACR Project GENIE Biopharma Collaborative in cBioPortal. Cancer Res..

[29] Győrffy B. (2024). Integrated Analysis of Public Datasets for the Discovery and Validation of Survival-Associated Genes in Solid Tumors. Innovation.

[30] Arafeh R., Shibue T., Dempster J.M., Hahn W.C., Vazquez F. (2025). The Present and Future of the Cancer Dependency Map. Nat. Rev. Cancer.

[31] Charitou T., Srihari S., Lynn M.A., Jarboui M.-A., Fasterius E., Moldovan M., Shirasawa S., Tsunoda T., Ueffing M., Xie J. (2019). Transcriptional and Metabolic Rewiring of Colorectal Cancer Cells Expressing the Oncogenic KRASG13D Mutation. Br. J. Cancer.

[32] Lee H., Kim B., Park J., Park S., Yoo G., Yum S., Kang W., Lee J.-M., Youn H., Youn B. (2025). Cancer Stem Cells: Landscape, Challenges and Emerging Therapeutic Innovations. Signal Transduct. Target. Ther..

[33] Abdou Hassan W., Muqresh M.A., Omar M. (2022). The Potential Role of CD44 and CD133 in Colorectal Stem Cell Cancer. Cureus.

[34] Wang C., Xie J., Guo J., Manning H.C., Gore J.C., Guo N. (2012). Evaluation of CD44 and CD133 as Cancer Stem Cell Markers for Colorectal Cancer. Oncol. Rep..

[35] Li P., Huang D. (2024). Targeting the JAK-STAT Pathway in Colorectal Cancer: Mechanisms, Clinical Implications, and Therapeutic Potential. Front. Cell Dev. Biol..

[36] Kahraman N., Biltekin E., Gul O.A., Kara G., Fokt I., Akay Y.M., Priebe W., Akay M., Ozpolat B. (2025). Abstract 6953: Inhibition of Eukaryotic Elongation Factor 2 Kinase (eEF2K) Disrupts Key Processes in Colorectal Cancer Progression and Tumor Growth. Cancer Res..

[37] Jin C., Wang T., Yang Y., Zhou P., Li J., Wu W., Lv X., Ma G., Wang A. (2023). Rational Targeting of Autophagy in Colorectal Cancer Therapy: From Molecular Interactions to Pharmacological Compounds. Environ. Res..

[38] Ghafouri Sabzevari F., Momeni-Moghaddam M., Farassati F., Rad A. (2016). Effect of EZH2 Inhibition on Colorectal Cancer Cells: An In Vitro Study. J. Genet. Resour..

[39] Kerkhove L., Geirnaert F., Rifi A.L., Law K.L., Gutiérrez A., Oudaert I., Corbet C., Gevaert T., Dufait I., De Ridder M. (2023). Repurposing Sulfasalazine as a Radiosensitizer in Hypoxic Human Colorectal Cancer. Cancers.

[40] Chaitanya G.V., Alexander J.S., Babu P.P. (2010). PARP-1 Cleavage Fragments: Signatures of Cell-Death Proteases in Neurodegeneration. Cell Commun. Signal..

[41] Zhang W., Liu Y., Liao Y., Zhu C., Zou Z. (2024). GPX4, Ferroptosis, and Diseases. Biomed. Pharmacother..

[42] Ma T., Du J., Zhang Y., Wang Y., Wang B., Zhang T. (2022). GPX4-Independent Ferroptosis—A New Strategy in Disease’s Therapy. Cell Death Discov..

[43] Jiang X., Stockwell B.R., Conrad M. (2021). Ferroptosis: Mechanisms, Biology and Role in Disease. Nat. Rev. Mol. Cell Biol..

[44] Biltekin E., Dilmac S., Kahraman N., Gul O.A., Akay Y.M., Wang Z., Akay M., Ozpolat B. (2026). Tumor-Suppressive microRNA Therapy Inhibits Growth of Glioblastoma Multiforme Xenografts. Cancers.

[45] Biltekin E., Kahraman N., Gul O.A., Akay Y.M., Akay M., Ozpolat B. (2025). Inhibition of FOXM1 Leads to Suppression of Cell Proliferation, Migration, and Invasion Through AXL/eEF2 Kinase Signaling and Induces Apoptosis and Ferroptosis in GBM Cells. Int. J. Mol. Sci..

[46] Mendieta M., Bandegi M., Biltekin E., Akay Y.M., Ozpolat B., Akay M. (2025). MiR 329/449 Suppresses Cell Proliferation, Migration and Synergistically Sensitizes GBM to TMZ by Inhibiting Src/FAK, NF-kB, and Cyclin D1 Activity. Int. J. Mol. Sci..

[47] Mendieta M., Avci N.G., Pandurangi R., Akay Y.M., Akay M. (2023). Targeted Sensitization of Glioblastoma Multiforme Using AAAPT Technology. IEEE Open J. Eng. Med. Biol..

